# Overexpressed Proteins in Hypervirulent Clade 8 and Clade 6 Strains of *Escherichia coli* O157:H7 Compared to *E*. *coli* O157:H7 EDL933 Clade 3 Strain

**DOI:** 10.1371/journal.pone.0166883

**Published:** 2016-11-23

**Authors:** Natalia Amigo, Qi Zhang, Ariel Amadio, Qunjie Zhang, Wanderson M. Silva, Baiyuan Cui, Zhongjian Chen, Mariano Larzabal, Jinlong Bei, Angel Cataldi

**Affiliations:** 1 Institute of Biotechnology, CICVyA, National Institute of Agricultural Technology. Hurlingham, Buenos Aires, Argentina; 2 AGRO-Biological Gene Research Center, Guangdong `Academy of Agricultural Sciences (GDAAS), Guangzhou, China; 3 Rafaela Experimental Station, National Institute of Agricultural Technology. Rafaela, Santa Fe, Argentina; Cornell University, UNITED STATES

## Abstract

*Escherichia coli* O157:H7 is responsible for severe diarrhea and hemolytic uremic syndrome (HUS), and predominantly affects children under 5 years. The major virulence traits are Shiga toxins, necessary to develop HUS and the Type III Secretion System (T3SS) through which bacteria translocate effector proteins directly into the host cell. By SNPs typing, *E*. *coli* O157:H7 was separated into nine different clades. Clade 8 and clade 6 strains were more frequently associated with severe disease and HUS. In this study, we aimed to identify differentially expressed proteins in two strains of *E*. *coli* O157:H7 (clade 8 and clade 6), obtained from cattle and compared them with the well characterized reference EDL933 strain (clade 3). Clade 8 and clade 6 strains show enhanced pathogenicity in a mouse model and virulence-related properties. Proteins were extracted and analyzed using the TMT-6plex labeling strategy associated with two dimensional liquid chromatography and mass spectrometry in tandem. We detected 2241 proteins in the cell extract and 1787 proteins in the culture supernatants. Attention was focused on the proteins related to virulence, overexpressed in clade 6 and 8 strains compared to EDL933 strain. The proteins relevant overexpressed in clade 8 strain were the curli protein CsgC, a transcriptional activator (PchE), phage proteins, Stx2, FlgM and FlgD, a dienelactone hydrolase, CheW and CheY, and the SPATE protease EspP. For clade 6 strain, a high overexpression of phage proteins was detected, mostly from Stx2 encoding phage, including Stx2, flagellin and the protease TagA, EDL933_p0016, dienelactone hydrolase, and Haemolysin A, amongst others with unknown function. Some of these proteins were analyzed by RT-qPCR to corroborate the proteomic data. Clade 6 and clade 8 strains showed enhanced transcription of 10 out of 12 genes compared to EDL933. These results may provide new insights in *E*. *coli* O157:H7 mechanisms of pathogenesis.

## Introduction

*Escherichia coli* O157:H7 is a human pathogen responsible for various diseases, including diarrhea, hemorrhagic colitis and hemolytic uremic syndrome (HUS). HUS is a disease whose incidence in industrialized countries such as the US, Canada and Japan, is one to three cases per 100,000 per year in children under 5 [1, 2]. Unfortunately Argentina is the country with the world's highest reported incidence, with about 14 cases per 100,000 in children under 5 and a report of 500 cases per year [3, 4]. Therefore, HUS is the leading cause of chronic and acute renal failure in children, causing 20% of kidney transplants in children and adolescents [5]. Herbivores are the main reservoir of Shiga toxin-producing *E*. *coli* (STEC). STEC and Enterohemorrhagic *E*. *coli* (EHEC) colonize a high percentage of domestic cattle in many countries but do not cause HUS in these animals [6–9].

EHEC is characterized for the presence of two major virulence factors, Shiga toxins and the Type Three Secretion System (T3SS) [10, 11]. The Shiga toxin (Stx), also denominated Verocytotoxin (VT), is the most relevant virulence factor in EHEC. Human infection starts when EHEC colonizes the large intestine and releases the Stx, which may be type 1 (Stx1) and/or type 2 (Stx2), and the latter have several subtypes, being any of these necessary for the development of HUS [12]. T3SS is encoded in a 35.6 kb pathogenicity island, which is called the locus of enterocyte effacement (LEE). EHEC uses T3SS to inject its own high affinity receptor Translocated intimin receptor (Tir) into the host cell. T3SS translocate several virulence factors into epithelial cells. These virulence factors, which are called effectors, manipulate the epithelial cell biology, thus favoring the bacteria.

EHEC O157:H7 isolates are genetically diverse according to different genotyping methods [13]. Using SNPs typing, Manning *et al* [14] separated *E*. *coli* O157:H7 into nine different clades. Among them, clade 8 strains had a strong association with a severe HUS disease [14] and it was initially related to the consumption of fresh spinach. The clade 8 strains were found in various clinical cases on multiple countries, including Argentina [14]. To date, the factors that define the hypervirulence of these strains are not completely understood. Most clade 8 strains have two subtypes of Stx2, Stx2a and Stx2c, with a higher expression of Stx2 than other clades [15]. Stx2a showed a greater association with HUS then Stx2c [16, 17]. Moreover, these strains have unique genetic features that may be relevant to causing the disease [18]. Iyoda e*t al* demonstrated a significant association not only between clade 8 strains and HUS cases but also with clade 6 strains [19].

Several groups have previously observed a predominance of clade 8 and clade 6 strains in cattle from different Argentinean provinces [20, 21]. On the other hand, a high prevalence of clade 8 strains was demonstrated in HUS patients in Argentina [22, 23].

Latest generation quantitative proteomics allows the simultaneous identification and assessment of the differential expression of thousands of proteins in different organisms. Our purpose was to identify differentially expressed proteins in two strains of *E*. *coli* O157:H7 from cattle and compared them to the well characterized EDL933 (clade 3) strain. These isolates from cattle, which belong to clade 8 and 6, showed enhanced pathogenicity in a mice model and virulence-related properties when compared to EDL933 [24]. Cell extracts and culture supernatants of strains Rafaela II (clade 8), 7.1 Anguil (clade 6) and EDL933 (clade 3) were prepared. Afterwards, we employed TMT-labeling and tandem two-dimensional liquid chromatography separation coupled with mass spectrometry (2D-LC MS/MS) to identify and quantify the differential expression of the proteins of the *E*. *coli* O157:H7 strains.

## Materials and Methods

### Bacteria and bacterial growth

Rafaela II (clade 8) and 7.1 Anguil (clade 6) are two *E*. *coli* O157:H7 strains isolated from cattle in the central humid Pampas, Argentina, in 2009. Both strains produced elevated levels of Shiga toxin 2 and had high lethality in mice [24]. The well characterized *E*. *coli* O157:H7 EDL933 (clade 3) strain recovered from a patient in USA was included in the study as a control, in all assays. The basis for the classification of Rafaela II, 7.1 Anguil and EDL933 as clade 8, clade 6, and clade 3, was described previously [24]. The bacteria were grown on Luria-Bertani (LB, Difco Laboratories, USA) agar plates or in LB broth aerobically at 37°C. For functional studies, the strains were grown in LB broth overnight with 150 rpm shaking, and then diluted 1/50 in Dulbecco’s modified Eagle’s medium (DMEM)-F12 nutrient mixture. They were finally grown in this DMEM-F12 medium to exponential phase (optical density (OD) at 600 nm of 0.6) at 37°C under a 5% CO_2_ atmosphere with 50 rpm shaking.

### Quantitative proteomics

#### Protein Extraction, Digestion, and TMT Labeling

Three biological replicates of bacterial cultures were centrifuged at 5000 rpm for 20 min at 4°C and the culture supernatant was filtered (0.22 μm filters). The cellular extracts were resuspended in ice-cold lysis buffer (50 mM Tris-HCl, pH 7.5, 25 mM NaCl, 5 mM DTT and 1 mM PMSF) and disrupted by three cycles of rapid freezing in liquid N_2_ and subsequent placed in boiling water to 100°C. The homogenate was centrifuged at 30,000 × g for 10 min. The filtered culture supernatant and the homogenate supernatant were precipitated with 5 volumes of ice-cold acetone, and then put at -20°C overnight. The protein pellets were resuspended in buffer containing 8 M Urea, 2 M Thiocarbamide and 200 mM tetraethylammonium bromide at pH 8.5. The protein concentrations were determined by the Bradford assay using BSA as a standard.

The proteins from the filtered culture supernatant and the cellular extracts of each strain were reduced with 200 mM Tris-(2-carboxyethyl)-phosphine and alkylated with 375 mM iodoacetamide. After digestion using trypsin, the samples were labeled with TMT reagents, using the following pairs of tags: 126 with 127, 128 with 129, 130 with 131 for the cell extract from Rafaela II, EDL933 and 7.1 Anguil, respectively. 127 with 130, 128 with 131, 126 with 129 for the supernatants from Rafaela II, EDL933 and 7.1 Anguil, respectively.

#### High pH Reverse Phase Fractionation

The labeled peptides were firstly subjected to Sep-Pak SPE cartridges (Waters) to remove salt ions. The hpRP chromatography was performed with Dionex UltiMate 3000 model on an Xterra MS C18 column (3.5 um, 2.1 × 150 mm, Waters). Then the sample were dissolved in buffer A (20 mM ammonium formate, pH 9.5) and eluted with a gradient of 10 to 45% buffer B (80% acetonitrile (ACN)/20% 20 mM NH_4_HCO_2_) in 30 min, followed by 45% to 90% buffer B in 10 min, and a 5-min hold at 90% buffer B. Forty-eight fractions collected at 1 min intervals were merged into 12 fractions.

#### Nano LC-MS/MS Analysis by Orbitrap Fusion

The nano LC MS/MS analysis was carried out using a Orbitrap Fusion tribrid (Thermo-Fisher Scientific, San Jose, CA) mass spectrometer with an UltiMate 3000 RSLC nano system (Thermo-Dionex, Sunnyvale, CA). Each fraction was injected onto a PepMap C18 trapping column (5 μm, 200 μm × 1 cm, Dionex) and separated on a PepMap C18 RP nano column (3 μm, 75 μm × 15 cm, Dionex), by a 135 min gradient from 4 to 36% ACN in 0.1% FA.

In positive ion mode, MS spectra were acquired across 350–1550 m/z scan mass range, at a resolution of 120000 in the orbitrap with the max injection time of 50 ms. Tandem mass spectra were recorded in high sensitivity mode (resolution >30000) and made by HCD at normalized collision energy of 40. Each cycle of data-dependent acquisition (DDA) mode selected the top10 most intense peaks for fragmentation. All data were acquired with Xcalibur 2.1 software (Thermo-Fisher Scientific).

#### Protein Identification and Quantitation

Both were carried out by Mascot (version 2.4.1, Matrix Science, Boston, MA) against the databases described below. One missed cleavage was allowed with fixed carbamidomethylation (Cys), fixed 6-plex TMT modifications on Lys and N-terminal amines and variable modifications of oxidation (Met), deamidation (Asn and Gln). The peptide and fragment mass tolerance values were set as 8 ppm and 20 millimass units (mmu), respectively. The target-decoy strategy [25] and the Mascot-integrated percolator calculation were applied to estimate the false discovery rate (FDR). Only peptides above "identity" were counted as identified; furthermore, to be confidently quantified, a protein must produce at least two unique peptides that generate a complete TMT reporter ion series.

MS/MS based peptide and protein identifications were validated via Scaffold (version Scaffold_4.4.3, Proteome Software Inc., Portland, OR). Peptide identifications were accepted when the peptide FDR is below 1.0%. Protein identifications were accepted when the protein FDR is below 1.0%, and at least two unique peptides could be quantified. The proteins that contained similar peptides and could not be differentiated based on MS/MS analysis alone were grouped to satisfy the principles of parsimony. The intensities of reporter ions for each valid spectrum were normalized. The reference channels were normalized to produce a 1:1 fold change. All normalization calculations were performed using medians. The differentially expressed proteins were chosen by fold change larger or smaller than 2.31 and Mann Whitney Test p<0.05.

### Gene data base construction

To identify exclusive genes present in TW14359 and EDL933, we compared reference genomes gene content through OrthoMCL methodology [26] implemented in GET_HOMOLOGUES [27] using an inflation parameter of 1.5. Clusters were parsed to obtain shared (core) and exclusive genes and databases were constructed with those sequences.

### Bioinformatics analysis

The identified proteins were analyzed using the following prediction tools: [28] CELLO v.2.5 to predict sub-cellular localization and EffectiveT3 v.2.0.1 [29] to predict proteins putatively exported through Type Three Secretion System (T3SS). Functional annotations were assigned by the COG database [30] and Blast2GO [31]. Protein sequences were analyzed by BLASTp, PFAM and UNIPROT. Operons were analyzed using DOORS^2^ 2.0 [32].

### Quantitative RT-PCR

The culture pellets (50 ml) from mid logarithmic phase cultures (OD_600nm_ 0.6) were resuspended in 1 ml of Trizol (Invitrogen) and the cells were lysed by pipetting. Then, 200 μl of chloroform was added and incubated for 15 min at room temperature. Tubes were centrifuged at 10,000g for 15 min at 4°C and the supernatant was extracted with 100 μl of chloroform and alcohol precipitated with 600 μl of isopropanol at 70°C overnight. The pellets were washed with 75% ethanol and resuspended in 50 μl of diethyl pyrocarbonate-treated water (Sigma Aldrich). The RNA samples were treated with DNase amplified grade (Invitrogen), run through agarose gels to check RNA integrity and used as templates in cDNA synthesis. cDNA was produced using either total RNA as a template. Briefly, total *E*. *coli* RNA (8 mg) was reverse transcribed with Superscript II (Invitrogen) using random hexamer oligonucleotides (Invitrogen) to prime cDNA synthesis.

DNA-free RNA (1 μg) was mixed with 50 ng of random primers (Invitrogen) in 20 μl of final volume and reverse transcribed to total cDNA with SuperScript II reverse transcriptase (Invitrogen) following the manufacturer’s instructions. Identical reactions lacking reverse transcriptase were also performed to confirm the absence of genomic DNA in all samples. Quantitative PCR was performed in a Step One plus real time thermocycler (Applied Biosystems) under standard cycling conditions: 1 cycle at 95°C for 10 min and 55 cycles at 95°C for 15 s and 60°C for 1 min. To ensure the specificity of the PCR products, we performed melting curve analysis by heating products from 65°C to 95°C, incrementing 0.5°C every 5 s while monitoring fluorescence. Specific oligonucleotides for selected genes were designed using primer 3 (http://bioinfo.ut.ee/primer3/), and employed at a final concentration of 300nM, by using Master Mix QuantiTect SYBR Green (Qiagen), 1 μl of template cDNA and the pairs of primers listed in S1 Table. Genomic DNA-based standard curves were used to determine the efficiencies of the genes target amplification by real-time PCR. qPCR data were analyzed using the 2–ΔΔCT method with efficiency correction. The amplification curves were studied using LinReg software [33]. The fold change was calculated using *rpoA* (Alpha subunit of RNA polymerase) and *serC* (phosphoserine aminotransferase) as reference genes [34, 35]. The final results and permutation statistical analysis were assessed with fgStatistic software (http://www.infostat.com.ar/?lang=en). Quantitative PCR procedure was designed according to MIQE general recommendations [36]. The results are presented as ratios calculated with the Relative expression software tool (REST@) application described by Pfaffl *et al*. [37], based on three biological replicates in triplicate.

## Results

### Bacterial growth

As a first step for the quantitative proteomics analysis, we incubated the three bacterial strains at 37°C in DMEM medium in 5% CO_2_ atmosphere, with shaking at 50 rpm. We selected this condition of growth because it induces the Type Three Secretion System (T3SS) and other virulence factors [38]. The growth curve and the count of CFU/ml was highly similar for the three strains EDL933 (clade 3, reference strain), Rafaela II (clade 8) and 7.1 Anguil (clade 6) (S1 Fig). This result indicates that in these conditions the three strains have a similar culture behavior.

### Gene data base

For the quantitative proteomics analysis itself, we constructed a gene database comprising all non-redundant translated genes of strains *E*. *coli* O157:H7 EDL933 (clade 3) and TW14359 (clade 8) by using OrthoMCL algorithm. In total, 5908 orthologous clusters were detected. From those, the core genome corresponded to 4544 clusters, and 529 were unique to TW14359 whereas 835 were unique to EDL933. Because the genome sequence of clade 8 strain (Rafaela II) and clade 6 strain (7.1 Anguil) are not complete, we used the sequence of the clade 8 TW14359 *E*. *coli* O157:H7 to construct the gene data base.‬‬

### Descriptive quantitative proteomics

We identified approximately 39% (2308/5908) of all proteins potentially synthesized by *E*. *coli* O157:H7. The MS/MS system acquired and identified 2241 (S2 Table) proteins in the cell extracts and 1787 proteins in the culture supernatants (S2 Table). A total of 67 proteins were only present in the culture supernatants. From these, 13.4% proteins are predicted as extracellular, and 34% as periplasmic (S3 Table). In total, we detected 2308 non redundant proteins. The high number of proteins detected in the culture supernatants may be due to lysis occurring during culture, handling or to production of outer membrane vesicles (OMVs) when the bacteria were cultured as was recently described [39].

Our work demonstrated the biochemical presence of 163 and 114 hypothetical proteins in the cell extracts and culture supernatants, respectively. These proteins should be reannotated as “unknown function” or any function assign.

### Comparative quantitative proteomics

The analysis of the differentially expressed proteins was focused on those proteins with a fold change (FC) log2>1.2 and with a p<0.05 in the Mann Whitney Test for the comparisons of Rafaela II/EDL933 and 7.1 Anguil/EDL933 proteins. Higher fold changes mean higher production of a given protein in hypervirulent lineages compared to the well characterized EDL933 strain that belongs to clade 3 (3 to 16 fold change). The number of proteins detected as overexpressed above a threshold of log2 1.2 were for Rafaela II: 33 (1.47%) and 55 (3.1%) proteins in the cell extract and culture supernatant, respectively. For 7.1 Anguil, the overexpressed proteins were 31 (1.38%) and 61 (3.41%) in the cell extract and culture supernatant, respectively. The percentage of overexpressed proteins respect to the total proteins detected either in cell extract or culture supernatant is shown between brackets.

**Fig 1:**
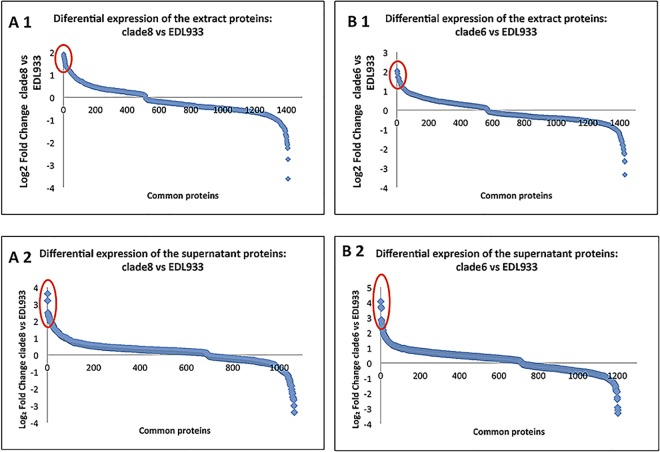
Differential expression of the proteins of cell extract (1) or culture supernatant (2) of *E*. *coli* O157:H7, evaluated by using the TMT-6plex labeling strategy associated with two-dimensional liquid chromatography and mass spectrometry (MS) in tandem. Results are shown as log2 Fold Change Rafaela II (clade 8) vs. EDL933 (A) or 7.1 Anguil (clade 6) vs. EDL933 (B) for each protein. Only proteins that p value <0.05 are presented. Proteins with a >1.2 fold change in each strain are indicated by a circle. Protein identification numbers correspond to S2 Table.

**Fig 2:**
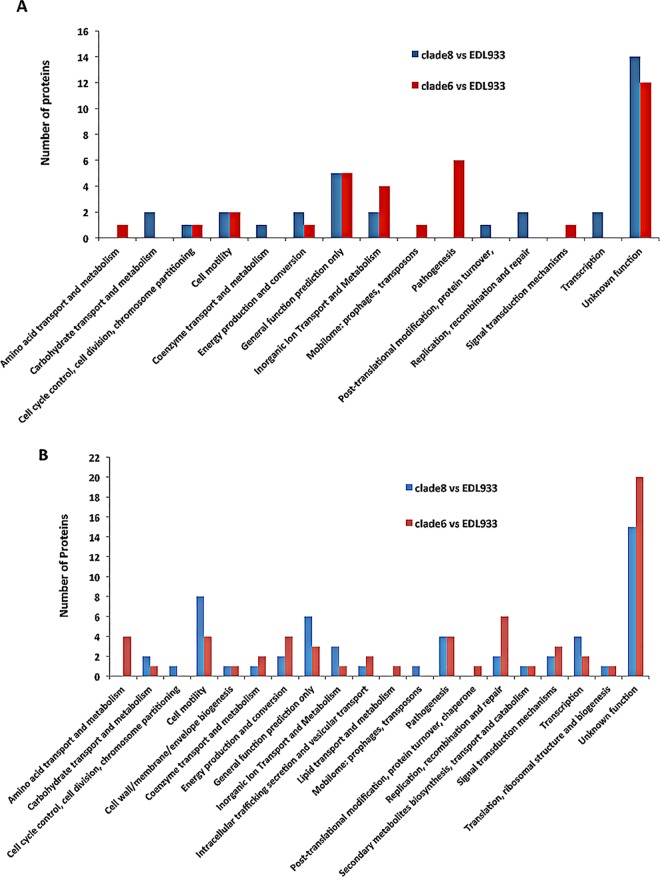
A, Functional categories of more abundant proteins identified in the cell extract of *E*. *coli* O157:H7 Rafaela II (clade 8) vs. EDL933 or 7.1 Anguil (clade 6) vs. EDL933. B, Functional categories of more abundant proteins identified in the culture supernatant of *E*. *coli* O157:H7 Rafaela II (clade 8) vs. EDL933 or 7.1 Anguil (clade 6) vs. EDL933.

The general picture of significant differential expression in Rafaela II vs. EDL933, and 7.1 Anguil vs. EDL933 is shown (Fig 1). For the Rafaela II vs. EDL comparison, we detected overexpression of proteins related to biological processes of unknown and general function, replication, transcription, recombination and repair, cell motility and carbohydrate transport in the cell extract (Fig 2A). For the 7.1 Anguil vs. EDL933 comparison, we found overexpression of proteins related to biological processes of unknown and general function, pathogenesis, aminoacid and ion transport and metabolism in the cell extract.

When comparing 7.1 Anguil and EDL933, we detected overexpression of proteins involved in biological processes of unknown function, cell motility, pathogenesis and transcription in the culture supernatant (Fig 2B). In comparing 7.1 Anguil to EDL933, we found overexpressed proteins related to biological processes of unknown function, replication, recombination and repair, pathogenesis, cell motility and aminoacid transport in the supernatants.

With these results in mind, we focused the following analyses on those proteins that are described or putatively related to virulence. We also studied proteins encoded in phages, because phages are associated to virulence [40, 41]. We also assessed other proteins that are described in the genome annotation as hypothetical. Among these proteins, we studied those with a high FC. Thus, we focused on the proteins with those characteristics: the top 16 proteins for the cell extracts and the top 12 proteins for the culture supernatants. We performed this analysis for both comparisons, Rafaela II vs. EDL933 and 7.1 Anguil vs. EDL933 (Table 1); more details of overexpressed proteins are given in S4 Table.

**Table 1:** Overexpressed proteins in E. coli O157:H7 Rafaela II (clade 8) and 7.1 Anguil (clade 6) compared to *E*. *coli* O157:H7 EDL933 (clade 3).

**Proteins Rafaela II (clade 8) cell extract**	**Fold change**	**P -value^a^**	**Proteins 7.1 Anguil (clade 6) cell extract**	**Fold change**	**P-value^a^**
PchE, like transcriptional activator [TW14359]^b^	3.76	0.021	Phage tail fiber protein EDL933_2453	4.1125	0.0001
YjbJ	3.56	0.00078	Protease TagA	3.9724	0.0001
CsgC, curli production protein	3.46	0.0039	EDL933_p0016	3.9724	0.00078
YebF, putative secreted protein (EDL933_2820)	3.43	0.0001	EDL933_1402	3.7064	0.0001
Phage protein EDL933_3226	3.29	0.021	Flagellin FliC	3.6808	0.0001
ECSP_5106 [TW14359] ^b^	3.25	0.021	General secretion pathway protein G EDL933_p0034	3.2944	0.00078
EDL933_0387	3.14	0.00078	Type III secretion protein EscF	3.2944	0.021
YicS, putative secreted protein (EDL933_4982)	3.12	0.0001	Conserved phage protein EDL933_1401	3.1383	0.0001
Phage protein ECSP_2742 [TW14359] ^b^	2.91	0.0001	Phage tail fiber protein EDL933_1406	3.1167	0.0001
Phage protein EDL933_1373	2.81	0.00078	DicA repressor ECSP_1724 [TW14359] ^b^	3.1167	0.0023
ECSP_1569 [TW14359] ^b^	2.66	0.0001	Stx2 subunit A	2.9079	0.0001
CheY, Chemotaxis regulator	2.62	0.00078	Phage protein EDL933_1388	2.8481	0.021
EDL933_3032	2.57	0.021	Membrane protein YijP	2.8089	0.0001
ECSP_1473 [TW14359] ^b^	2.51	0.00078	Dienelactone hydrolase	2.7511	0.0001
Serine protein kinase YeaG	2.51	0.0001	Haemolysin A (HlyA)	2.7511	0.0001
EDL933_p0089	2.40	0.00078	Serine protease autotransporter enterotoxin EspP	2.7511	0.0001
**Proteins Rafaela II (clade 8) culture supernatant**	**Fold change**	**P-value**^**a**^	**Proteins 7.1 Anguil (clade 6) culture supernatant**	**Fold change**	**P-value**^**a**^
Stx2a subunit B	12.30	0.0001	Phage protein EDL933_1403	16.450	0.0001
Phage protein EDL933_3254	9.25	0.0001	Stx2a subunit B	12.996	0.0001
Regulator of flagellin synthesis FlgM	5.66	0.0001	EDL933_1400	12.295	0.0001
Phage protein EDL933_1785	5.35	0.0001	Dienelactone hydrolase	7.160	0.0001
Phage tail fiber protein EDL933_2012	5.10	0.0039	Phage protein EDL933_1385	6.727	0.0001
Phage protein EDL933_1726	5.03	0.0001	Conserved phage protein EDL933_1401	6.105	0.0001
Dienelactone hydrolase	4.69	0.0001	EDL933_1410	5.696	0.0001
CheW, adaptor of CheA kinase	4.63	0.0001	EDL933_1419	5.205	0.0039
EDL933_p0016	4.59	0.0001	Phage tail fiber protein EDL933_1406	5.169	0.0001
FlgD, flagellar basal-body rod modification protein	4.29	0.0039	Phage protein EDL933_1399	5.063	0.0001
Serine protease autotransporter enterotoxin EspP	4.26	0.0001	Protease TagA	4.408	0.0001
CheY, chemotaxis regulator	4.08	0.00078	Haemolysin A (HlyA)	3.434	0.0001

^a^ p values were calculated using Mann-Whitney test.

^b^ b proteins identified in *E*. *coli* O157:H7 str TW14359.

### Overexpressed proteins in Rafaela II (clade 8) strain

In the cell extract the following are overexpressed (Table 1): the putative curli production protein CsgC, which is a small periplasmic chaperone that binds to curlin and prevents curli formation in the cell prior to export [42]; a PchE-like transcriptional activator, present in clade 8 TW 14359 strain that shares 90% identity with PchE activator of Sakai strain [43] and to a non-annotated ortholog in EDL933; yjbJ, that belongs to the CsbD family protein; and to bacterial general stress response protein [44]. In addition, six phage proteins were overexpressed. Two proteins described as hypothetical proteins in databases, ECSP_5106 present in clade 8 and present but not annotated in EDL933 and Sakai; and EDL933_0387 are also overexpressed; as well as two putative exported proteins, YebF and YicS. YebF was found in the cell extract of the three stains tested in this study (S2 Table), but has been described as secreted to the extracellular medium [45]. In turn, YicS (EDL933_4982) belonging to the family of proteins probably implicated in transport is also overexpressed. The eukaryotic like serine protein kinase, YeaG or stress kinase involved in stress adaptation of *E*. *coli* [46] is overexpressed. YeaG is highly conserved in other enterobacteria and contains AAA+ (ATPases Associated with diverse cellular Activities) and a *P*-*loop* NTPase domain. Other proteins are shown in Table 1.

In the culture supernatant, the following proteins are overexpressed (Table 1): Shiga toxin 2 subunit B corresponding to Stx2a, and a negative regulator of flagellin synthesis FlgM, which is an anti-sigma 28 factor of FlgI sigma factor [47] playing a central role in the control of flagellar organella of the bacteria. Rafaela II also overexpresses several phage proteins. Among those phage proteins EDL933_1726 is interesting because it has 645 aa identical to a 9-O-acetyl-N-acetylneuraminic acid deacetylase YjhS (NanS) of *E*. *coli*. Bacteria possess different enzymes that can exploit sialic acid as a fermentable carbon source. One of these enzymes is NanS, a carbohydrate esterase that deacetylates the 9 position of 9-*O*-sialic acid so that it can be readily transported into the cell for catabolism [48]. The pO157 plasmid encoded for the overexpressed proteins dienelactone hydrolase, which catalyzes the hydrolysis of dienelactone to maleylacetate, which plays a key role for the microbial degradation of chloroaromatics via chlorocatechols. The pO157 plasmid also encoded for EspP and EDL933_p0016, which is a Cytochrom_B562 superfamily member that is overexpressed in the cell extract of 7.1 Anguil strain. Some flagellar and chemotactic related proteins were also identified. Other proteins related to motility are shown in Table 1.

### Overexpressed proteins in 7.1 Anguil (clade 6) strain

The overexpressed proteins detected in the cell extract are the following (Table 1): several phage proteins and Shiga toxin2 A subunit must be considered also a phage protein. However, as the A subunits of toxins Stx2a and Stx2c are identical, we could not distinguish if it is expressed from the *stx2a* or the *stx2c* gene. The pO157 plasmid encoded for the overexpressed proteins TagA or StcE zinc-metalloprotease with 63% conserved aminoacid homology to the lipoprotein TagA from *Vibrio cholera*, a protease that degrades mucin. The plasmid encoded General secretion pathway protein G, described as the unique Type Two Secretion System in EHEC [49]; EDL933_p0016 and dienelactone hydrolase, which was also identified as overexpressed in Rafaela II strain. Finally, pO157 also encoded for the haemolysin (HlyA) belonging to the RTX (repeats in toxins) family of toxins and SPATE EspP. Other proteins overexpressed are shown in Table 1, especially interesting is YijP proposed as modifying the lipid A of LPS by a phosphoethanolamine moiety. This modification allows *E*. *coli* to cross the blood-brain barrier [50].

In the culture supernatant, the following proteins are overexpressed (Table 1): several proteins encoded in Stx2a phage and Shiga toxin 2a subunit B, as described for Rafaela II. The overexpressed proteins encoded for pO157 plasmid are dienelactone hydrolase, TagA or StcE zinc-metalloprotease, which is detected as overexpressed in the cell extract of the same strain and haemolysin (HlyA).

### Promoter and intergenic region analysis

To assess if changes in protein expression levels were associated to promoter changes, an analysis of intergenic regions (IR) upstream of genes shown in Table 1 was performed by *in silico* comparing TW14359 and EDL933 strains. It was assumed that the promoters must be contained in the intergenic region located between start codon of the gene encoding for the protein overexpressed (when monocistronic) or the first operon gene (when is part of an operon) and the stop codon of gene upstream. First it was needed to determine if the genes encoding for overexpressed proteins described in Table 1 are part of an operon. For that, Doors 2 software was used. In most of the cases (47/56) the intergenic regions (IR) were 100% conserved between clade 8 TW14359 and clade 3 EDL933 strain. The 8 IR with differences are described below and in Table 2. In the case of IR of Phage protein EDL933_1373 there is an A/G substitution 25 nt upstream of the first gene of the corresponding operon. In the case of stx2a, the Q antiterminator protein and the intergenic region between Q antiterminator protein gene and stx2a gene were analyzed. There are no substitutions neither in the Q gene having both strains the q933 allele, but there are two substitutions and a 2 nt deletion in late promoter pR´and tRNA genes region of TW14359 compared to EDL933. For EDL933_3254 there is one substitution in middle of the intergenic region. In the case of EDL933_1785 there are several substitutions in the intergenic region upstream of the gene (twelve 1 nt, two 2 nt, one 3 nt and one case of 1 nt insertion in TW14359). For EDL933_1726, EDL933_1385 and EDL933_1419 there are 5, 4, and 4 substitutions of 1 nt respectively. The activator pchE like (ECSP_1500) gene has a non-annotated 93% conserved ortholog in EDL933, but the intergenic sequences have no significant homology. As DicA repressor and Phage protein ECSP_2742 have no orthologues in EDL933.

**Table 2:** Analysis of intergenic regions (IR) upstream of overexpressed-proteins genes in *E*. *coli* O157:H7 Rafaela II (clade 8) and 7.1 Anguil (clade 6). Only those IR showing sequence variability between *E*. *coli* O157:H7 are described.

gene	variation in TW14359 respect to EDL933^a^	operon	position in operon	comment
PchE like	no significant homology	yes	other^b^	
Phage protein EDL933_1373	69/70(99%)	yes	other	1 s^c^
Stx2a (Q protein)	435/435(100%)	yes	first gene	
Stx2a (region pR´tRNAs)	779/783(99%)			2 s, 1 d
EDL933_3254	130/131(99%)	yes	other	1 s
EDL933_1785	385/406(95%)	no	other	15 s, 1 i
Phage protein EDL933_1726	230/235(98%)	yes	other	5 s
Phage protein EDL933_1385	482/486(99%)	yes	first gene	4 s
EDL933_1419	89/94(95%)	yes	other	4 s, 1 i

^a^ number of identical nt/length of the intergenic region

^b^ position other than the first

^c^ s, substitution, d, deletion, i, insertion

### Gene expression quantification of Rafaela II (clade 8) and 7.1 Anguil (clade 6) strains by RT- qPCR

**Fig 3:**
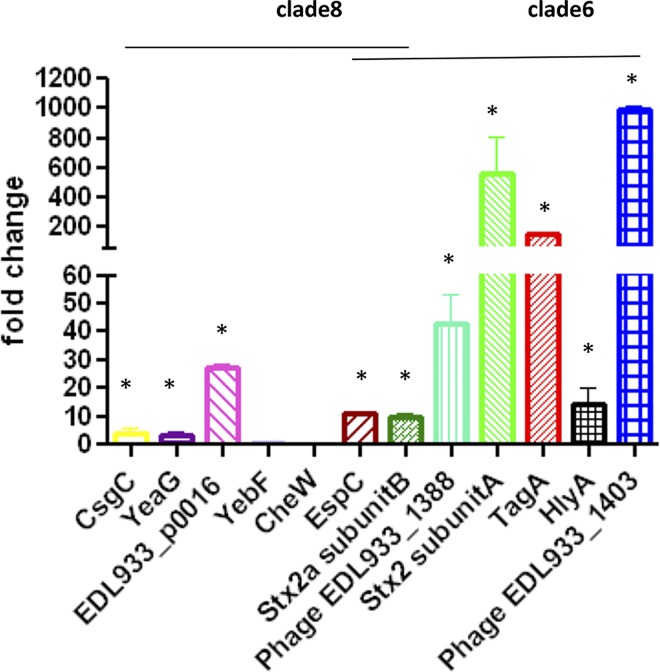
Comparison of the differential (clade 6/8 strain vs. EDL933) gene expression ratios of selected genes of *E*. *coli* O157:H7, obtained by RT-qPCR of RNA from bacteria grown in vitro. The bars indicate the fold change of Rafaela II (clade 8)/EDL933 or 7.1 Anguil (clade 6)/EDL933 for three independent biological replicates, for triplicate, and the error bars indicate the standard deviations. The values of gene expression in clade 8 or clade 6 strain were significantly different of those in EDL933, as determined by fgStatistic software(* p value < 0.05).

Proteins whose expression varied between the studied strains were validated by RT-qPCR in a subset of 12 genes (Fig 3). We used two genes, *rpoA* and *serC* as references. We found that the proteins CsgC, PrkA, Phage EDL933_1388, TagA, Stx2 subunitA, Stx2a subunitB, HlyA, EDL933_p0016, Phage EDL933_1403 and EspP are differentially expressed between the virulent *E*. *coli* strain Rafaela II and 7.1 Anguil compared to the EDL933 strain, with mRNA expression fold changes varying from 2.86 (yeaG) to 946.27 (EDL933_1403). All of these results have a p-value <0.05. Thus, the concordance between the proteomics results and the RT-qPCR results was acceptable, supporting the proteomic approach used in this study. On the other hand, we could not confirm the differential expression of YebF and CheW that was detected in the proteomics experiments by RT-qPCR. However, RT-qPCR is not always the right validation for quantitative proteomics. A protein could be overexpressed due to post transcriptional regulation (i.e. by ncRNA) or can be exported in higher quantities because of an up regulation of a secretion system.

## Discussion

In this study we defined the quantitative differential proteome of two hypervirulent *E*. *coli* O157:H7 Rafaela II (clade 8) and 7.1 Anguil (clade 6) strains compared to the well characterized reference *E*. *coli* O157:H7 EDL933 (clade 3) strain.

The isobaric tag labeling approach is powerful in providing biologically meaningful clues in comparative pathogen proteomics, when executed on high-performance hybrid orbitrap mass spectrometers [51]. The depth of proteome coverage affects quantitative analysis as well. Hence, appropriate prefractionation was applied to reduce the peptides complexity in fractions, which were analyzed one by one on MS. Then yields of peptide-spectrum matches (PSMs), unique peptides and proteins were enhanced. Because the peptides were more possible to be detected, since less candidates appeared in most scans. In this study, we applied hpRP liquid chromatography separation to the samples after TMT-6plex labeling. Indeed, hpRP-LC provides better resolution compared with several other LC-compatible methods, e.g. off-gel electrophoresis, strong cation exchange [52, 53].

In this study, we identified 2241 quantifiable EHEC cytosol proteins via Obitrap Fusion. Although the amount of protein IDs is almost two times as large as the amount for *E*. *coli* MC4100 [54], it is a bit smaller than the amount (>2300 label-free quantifiable proteins) for *E*. *coli* BW25113 in the present report [55]. This minor loss may mainly due to different factors. For instance, not all peptides may have been labeled successfully. Unlabeled peptides may have occupied MS time and therefore may have not contributed to protein IDs, since TMT-labeling had been set as fixed modification in the database search. Label-free quantification detours such circumstance at the cost of more frequent consideration of LC run-to-run repeatability. Our work identified a high number of proteins in the culture supernatants of *E*. *coli* O157:H7, much higher than expected from the number of *E*. *coli* proteins having signal sequence or from the analysis of 1D or 2D gels stained with silver nitrate. This abundance of *E*. *coli* extracellular proteins was already observed by other groups working with MS proteomics and the possible mechanisms were recently discussed [39].

In all analyzed relationships most overexpressed proteins are those of unknown function. This result can be explained by the fact that many *E*. *coli* O157: H7 proteins are annotated as hypothetical proteins. Our work demonstrated the biochemical presence of these proteins that should be reannotated as “unknown function”. In some cases we were able to identify probable functions based on similarity or conserved domains identification (data not shown). We preselected proteins with a clear relationship with virulence and also phage proteins because in EHEC many virulence proteins are encoded in phages. However, for many proteins there was no evident relationship with virulence. This is case of many phage proteins.

Some of the proteins identified here as overexpressed are related by genetic origin or by biochemical function; the others are individually presented.

Lambdoid phage encoded proteins are the main family of overexpressed proteins. Some of the highest expressed phage genes, especially in the 7.1 Anguil strain, were those encoded in BP-933stx2, which encodes for the Stx2 Shiga toxin. The proteins EDL933_1373; EDL933_1385; EDL933_1388; EDL933_1399; EDL933_1400, EDL933_1401; EDL933_1402; EDL933_1403; EDL933_1406; EDL933_1410 and EDL933_1419 were overexpressed and the genes maps downstream of Stx2 gene in the Stx2a encoding phage. Indeed, Kulasekara et al [18] demonstrated the relevance of the proteins related to virulence and encoded in phages in clade 8 strains. In this study, however, these proteins were more overexpressed by clade 6 strain than clade 8 strain. The mechanism of enhanced expression of Stx2a has been proposed to be influenced by the allele of Q antiterminator gene present in the Stx2a encoding phage [40, 56]. In accordance, the expression of the genes downstream Stx2 genes should be also controlled by Q and the late promoter as all are encoded in the same chain. In addition it was demonstrated that the expression level of Stx2 is not only regulated by the element of Q gene, but also by Stx2 promoter, RBS and by regulators located outside the phage [57, 58]. It has been reported that genes of the Stx2 phage are constitutively expressed even without lysis induction [59](Maite Muniesa, personal communication). We found many of Stx2a phage proteins in the culture supernatant of 7.1 Anguil strain. A probable explanation is that 7.1 Anguil suffers from a higher lysis provoked by Stx2a phage (or another phage) compared to EDL933 strain. However, this differential lysis may not be detectable by CFU counting. Nonetheless, when the presence of ribosomal proteins in culture supernatants, that may be considered a marker of bacterial lysis, was examined, a higher presence of ribosomal proteins in extracellular media in strain 7.1 Anguil was not observed. For the 65 ribosomal proteins detected, the fold change was 0.3±0.4 (data not shown). Another possibility is that lysis level is the same between the strains but that the expression level of Stx2a leaded by late promoter is stronger. It is important to consider that Stx2 phage are constitutively expressed even without lysis induction. For that it must be considered heterogeneity in phage induction and the existence of a mixed population of lytic and lysogenic phages [59].

Overexpressed EDL933_2012 is encoded in the phage CP933O/Sp4, as well as the effectors EspF2-1 and EspV are also encoded in this phage [40], CP-933C phage encodes for the 9kDa EDL933_1785 and the PchE activator. These proteins are overexpressed in the clade 8 strain and much less in the clade 6 strain (FC 1.71). The Pch regulators are present only in EHEC, and are functional analogues to the Per activators of atypical EPEC. The *per* operon genes are located in a 90 kb plasmid (EAF plasmid) in EPEC. This operon regulates the expression of LEE pathogenicity island and fimbriation [60, 61]. In contrast to EPEC, EHEC which lacks pEAF plasmid has a family of functional prophage-encoded PerC homologous proteins, which are called PchA to E. [43, 62]. Importantly, genomic variations in regions adjacent to *pch* are associated to diversity in expression patterns of the LEE [62]. CP-933V/Sp15 encodes for EDL933_3254 and Sp9 for EDL933_2453, which are overexpressed in clade 6 strain. Relevantly, this prophage encodes for eight Nle effectors. CP-933U/Sp14 prophage encodes for the overexpressed EDL933_3226, EDL933_3032 and also for the effector TccP, which is exclusive of EHEC. ECSP_1569 belonging to the HipB regulator superfamily is located in the Sp8 phage in *E*. *coli* O157:H7 Sakai strain. Interestingly, the gene of this regulator is interrupted by an integrase in the EDL933 strain. Finally, CP-933M/Sp6, which encodes for the effectors EspX7, EspN, NleB2-2, EspO1-1, overexpresses EDL933_1726 in clade 8 strain. This protein has a domain of deacetylase of sialic acid, and has a sequence alteration between clade 8 strain and EDL933. It was recently published that *E*. *coli* O157:H7 posses several paralogs of nanS, many of them encoded in prophages with diverse specificity for sialic acid forms [63, 64]. Those sialidases could be released upon bacterial lysis provoked by the Stx encoding phages. The hypothetical regulator ECSP_1473 is also encoded in this phage.

Shiga toxins are also encoded in prophages. Stx2 was previously observed as overexpressed in the clade 8 strain TW14359 [65]. In addition, in a previous work, we observed that 7.1 Anguil, the clade 6 strain, was the most cytotoxic for Vero Cell lines and also the strain that produces more Shiga toxin, evidenced by ELISA [24]. The clade 8 strain expresses more Stx2a than the Stx2c variant [18]. The factors that contribute to this enhanced expression of the Stx2 genes are not clearly understood. However, we observed overexpression in many Stx2a phage genes in clade 6 strain. In supernatants from both clades, the MS/MS assigned the B subunit to the Stx2a type, in line with Kulasekara et al. findings [18].It is highly probable that increased production of Stx2 contributes to the increased lethality of Rafaella II and 7.1 Anguil we previously observed [24].

The plasmid pO157 encodes for several of the overexpressed proteins in both strains. EspP is a SPATE type secreted protease and its role in virulence has been well determined for EPEC [66] but much less for EHEC. Indeed, in EHEC, EspP regulates pore formation and cytotoxicity mediated by the Type III Secretion System [67]. The haemolysin (HlyA) belongs to the RTX family of toxins and is encoded in the pO157 plasmid [68]. No mutant has been reported for the HlyA gene and its role in pathogenesis is not clear yet. Less studied are the pO157 encoded protein TagA and dienelactone hydrolase. TagA or StcE is an extracellular zinc-metalloprotease that degrades mucin in *Vibrio cholera* [69]. This protein was identified as antigenic in HUS patients [70]. The presence of a dienelactone hydrolase is intriguing as in *Pseudomonas* sp. this enzyme is part of the chlorocatechols degradation pathway. Chlorocatechols are organic pollutant in the environment. It is possible to speculate that this enzyme may be involved in the sensing and utilization of the hormones epinephrine and norepinephrine, which are catecholamines [71]. Relevantly, the General secretion pathway protein G is overexpressed in clade 6 strain and represents the only case in which a Type Two Secretion System is encoded in a plasmid and not in the genome [49]. It has been demonstrated that a mutant in Type Two Secretion System encoded in pO157 affect the colonization capacity of the bacteria to epithelial cells and intestinal colonization [72].

In previous work we observed that Rafaela II (clade 8) and 7.1 Anguil (clade 6) are highly virulent in various *in vitro* assays (adherence, Shiga toxin activity and RBC lysis) and in murine lethality. One of these tests, RBC lysis is related to T3SS activity and, in spite of the fact that there is variability in strains of the same clade; one may expect more abundant T3SS proteins in clade 6 and 8 strains. However the contents of EspA, B and D are higher in *E*. *coli* EDL933 than in clade 6 and 8 strains (data not shown). To explain this paradox one may speculate that T3SS proteins are not the only factor to contribute to RBC lysis and that non-T3SS adhesins are important for the attachment to erythrocytes. Also proteomics quantitation does not measure formed T3SS but proteins components of T3SS that may be soluble and not multimerized on the secretion system. Microscopy may be a better way to measure T3SS per bacterial cell.

In addition, the chemotactic-flagellar system is represented among the overexpressed proteins by two regulators (FlgM and FlgD). Notably, flagellin is overexpressed in clade 6 strain, whereas FlgM is overexpressed in clade 8 strain. FlgM is an anti-sigma 28 factor of FlgI sigma factor [47] that activates flagellin synthesis. These overexpressions seem to be contradictory and require further investigation. Also, the FlgD flagellar basal-body rod modification protein and CheY are overexpressed by clade 6 and 8 strains. CheY is a regulator that relays information between methyl-accepting chemoreceptors and the flagellar motor switch [73]. CheW (overexpressed in clade 8 strain) is an adaptor protein that binds to CheA kinase and to the methyl-accepting chemoreceptors. Surprisingly, we detected FlgM in culture supernatant. However, this protein has been reported as secreted in *Salmonella sp*. [74]. Another surface appendage that is overexpressed is the CsgC, a curlin chaperone [75]. Curli contributes to virulence by adherence and biofilm formation in epithelial cells and abiotic surfaces [76].

In general, the cause of overexpression is not understood leading us to perform an analysis on intergenic regions upstream of genes of protein overexpressed comparing strains *E*. *coli* TW14359 and EDL933. As most of the promoters of these proteins are not mapped we assumed that promoters must be included in these intergenic regions. Most of the intergenic regions of proteins shown in Table 1 are identical between the compared strains. Eight of them showed sequence variations, but at the moment it is not possible to conclude that these changes are the cause of enhanced expression. In most of the cases, the IR sequences are identical between the two strains and probably the identification of several regulator genes overexpressed in clade 6 and clade 8 strains may be a clue for this enhanced expression.

This work allows us to associate descriptive proteomics results with previous findings of virulence and pathogenicity in *in vitro* models and *in vivo* models, in mouse. This work provides promising new candidate proteins to be investigated in *E*. *coli* O157:H7 infections. Further studies in these proteins and genes could contribute to the definition of antimicrobial strategies used for enterohemorrhagic *E*. *coli*.

## Supporting Information

S1 Fig Growth curve of *E*. *coli* O157:H7 in DMEM media. Bacterial strains EDL933, Rafaela II (clade 8) and 7.1 Anguil (clade 6) were grown in LB broth overnight at shaking at 150 rpm and then diluted 1/50 in Dulbecco’s modified Eagle’s medium (DMEM)-F12 medium and grown at 37°C under a 5% CO_2_ atmosphere with shaking at 50 rpm. Results are shown as A: OD at 600nm per time in hours and B: CFU/ml per time in hours.
(TIF)

S1 Table docx: Primers used for RT-qPCR. (DOCX)

S2 Table Dataset of all proteins detected- There are a sheet for cell extract an another for culture supernatant. (XLSX)

S3 Table Proteins found only in culture supernatant. (XLSX)

S4 Table Overexpressed proteins. Top Fold Change proteins for cell extract (n = 16) and culture supernatant (n = 12). This table have complementary data respect to Table 1 in the manuscript.
(XLSX)
