# Supplementary figures and images for: Overexpressed Proteins in Hypervirulent Clade 8 and Clade 6 Strains of Escherichia coli O157:H7 Compared to E. coli O157:H7 EDL933 Clade 3 Strain
Source: PLoS One. 2016 Nov 23;11(11):e0166883. doi: 10.1371/journal.pone.0166883 (PMC5120812; doi:10.1371/journal.pone.0166883)

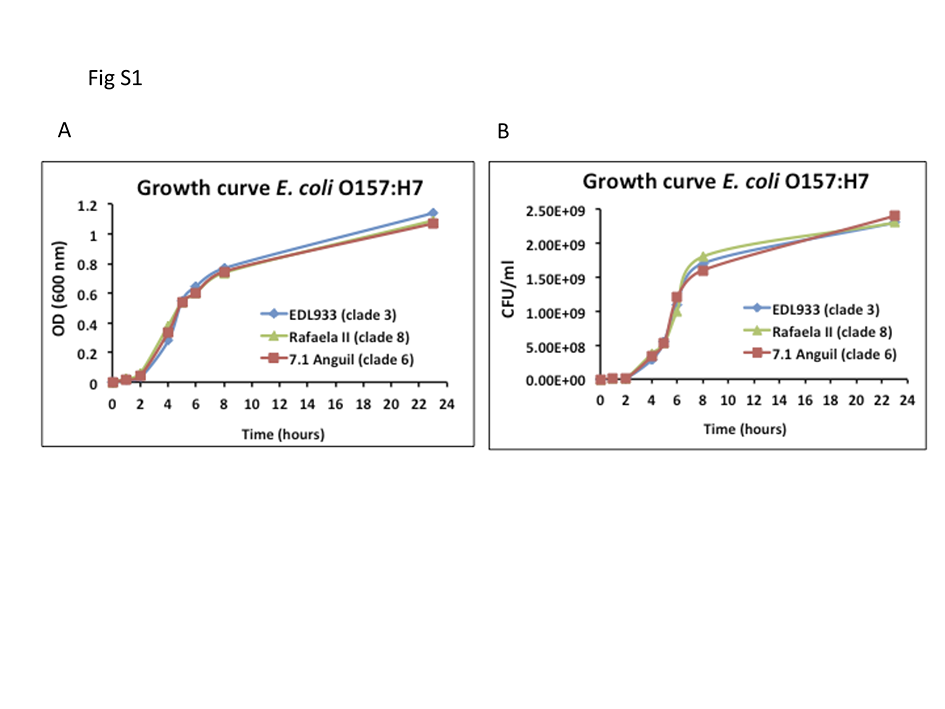

Supplement: S1 Fig — Bacterial strains EDL933, Rafaela II (clade 8) and 7.1 Anguil (clade 6) were grown in LB broth overnight at shaking at 150 rpm and then diluted 1/50 in Dulbecco’s modified Eagle’s medium (DMEM)-F12 medium and grown at 37°C under a 5% CO2 atmosphere with shaking at 50 rpm. Results are shown as A: OD at 600nm per time in hours and B: CFU/ml per time in hours. (TIF) [file pone.0166883.s001.tif]
