# Supplementary material for: Overexpressed Proteins in Hypervirulent Clade 8 and Clade 6 Strains of Escherichia coli O157:H7 Compared to E. coli O157:H7 EDL933 Clade 3 Strain
Source: PLoS One. 2016 Nov 23;11(11):e0166883. doi: 10.1371/journal.pone.0166883 (PMC5120812; doi:10.1371/journal.pone.0166883)
Supplement: S1 Table — (DOCX) [file pone.0166883.s002.docx]

Supporting Table 1 Primers used for RT-qPCR

| **Primer** | **Sequence** | **Gene or locus tag** | **Tm (C°)** | **amplicon size (bp)** | **References** |
| --- | --- | --- | --- | --- | --- |
| CsgC_F | ACCCAGCAAGGGGATATGTA | *csgC* | 59.2 | 112 | This study |
| CsgC_R | TCTGACTTTGCCCTGAACTG |  |  |  |  |
| yebF_F | GGGTCAGTTTGCAGGACATT | *yebF* | 60 | 101 | This study |
| yebF_R | GTCCACGCTGACCTGGTAAT |  |  |  |  |
| yeaG_F | GGTCAACGATCATCCGTTCT | *yeaG* | 60 | 146 | This study |
| yeaG_R | TGATATCGCCACCAAATTCA |  |  |  |  |
| EDL933_1388_F | CGTCTTGATGCAGAAGTGGA | *EDL933_1388* | 60 | 106 | This study |
| EDL933_1388_R | GCTGAACTTCACCGCTCTCT |  |  |  |  |
| TagA_F | GCTAAAGCGAAAGTGCTGCT | *tagA* | 60 | 119 | This study |
| TagA_R | TACTGTCCGTTCCAGGCTTT |  |  |  |  |
| Stx2subA_F | TATATCAGTGCCCGGTGTGA | Stx2A | 60 | 108 | This study |
| Stx2subA_R | TGACGACTGATTTGCATTCC |  |  |  |  |
| hlyA_F | AAGCCGGAACAGTTCTCTCA | hlyA | 60 | 101 | This study |
| hlyA_R | ATCCTCTCCTTCCCGTTGTT |  |  |  |  |
| Stx2asubB_F | ATGGCGGTTTTATTTGCATT | *Stx2B* | 59.3 | 121 | This study |
| Stx2asubB_R | CTTTCCCGTCAACCTTCACT |  |  |  |  |
| EDL933_p0016_F | GCGTCTTCCTGAGAGCATTT | *EDL933_p0016* | 60 | 105 | This study |
| EDL933_p0016_R | ACTTTTTGTTTCGCCAGCAT |  |  |  |  |
| EspC_F | GATGTCCGAAAAACCCAGAA | *espC* | 60 | 116 | This study |
| EspC_R | AGTCGGACAGTTCCATCACC |  |  |  |  |
| CheW_F | GTAACACGGATTGCGAACAC | *cheW* | 59.6 | 107 | This study |
| CheW_R | ACATCCACCTGGCTGAACTT |  |  |  |  |
| EDL933_1403_F | CCGTGAAAAAGGACACCATT | *EDL933_1403* | 60 | 123 | This study |
| EDL933_1403_R | CTTCATGACGAACCCAGACA |  |  |  |  |
| EDL933_1400_F | GAACAGGAGCAGGCAGTACC | *EDL933_1400* | 60 | 119 | This study |
| EDL933_1400_R | ATAATCGCCTCTGCCTCTGA |  |  |  |  |
| rpoA_F | GCGCTCATCTTCTTCCGAAT | *rpoA* | 60 | 200 | Gruber et al |
| rpoA_R | CGCGGTCGTGGTTATGTG |  |  |  |  |
| serC_F | AAAGCGAATGTCGCGTGTCC | *serC* | 60 | 200 | Herold et al |
| serC_R | CAATTTGTCAAGCGCACTGTCC |  |  |  |  |
